# Death of a Resident Medical Officer at Croydon

**Published:** 1911-09-30

**Authors:** 


### Death of a Resident Medical Officer at Croydon.

The death is announced at the early age of thirty-three of Dr. Thomas Augustine Dillon, the Assistant Resident Medical Superintendent of the Croydon Union Infirmary, Thornton Heath. Dr. Dillon took his medical studies in Dublin, and obtained the licentiates of both Royal Colleges in 1902. He held the appointments of House Physician and House Surgeon at St. Vincent's Hospital, Dublin, and was also Demonstrator of Anatomy at the Royal College of Surgeons of Ireland, of which he became a Fellow in 1905.

